# Comparative Study of Reprogramming Efficiency and Regulatory Mechanisms of Placental- and Fibroblast-Derived Induced Pluripotent Stem Cells (iPSCs) in Mules

**DOI:** 10.3390/cimb47080671

**Published:** 2025-08-19

**Authors:** Fangyuan Liu, Jia Zhang, Lingyu Kong, Rihan Wu, Qiqi Jiang, Ying Lu, Xihe Li

**Affiliations:** 1 State Key Laboratory of Reproductive Regulation & Breeding of Grassland Livestock, School of Life Sciences, Inner Mongolia University, Hohhot 010070, China; liufy@mail.imu.edu.cn (F.L.); zhangjia0922@126.com (J.Z.); wrh13074789040@126.com (R.W.); 22308064@mail.imu.edu.cn (Q.J.); 2 Research Center for Animal Genetic Resources of Mongolia·Plateau, College of Life Sciences, Inner Mongolia University, Hohhot 010070, China; 3 Inner Mongolia SaiKexing, Institute of Breeding and Reproductive Biotechnology in Domestic Animal, Hohhot 011517, China; 4 Liangzhu Laboratory, Zhejiang University Medical Center, Hangzhou 311113, China; 5 College of Fisheries and Life Science, Shanghai Ocean University, Shanghai 201306, China; m230100105@st.shou.edu.cn; 6 National Center of Technology Innovation for Dairy Industry, Hohhot 010020, China

**Keywords:** iPSCs, ASE, reprogramming, PI3K-AKT, interspecies hybrid, pluripotency

## Abstract

As an interspecies hybrid inheriting genetic material from horse and donkey lineages, mules provide a unique model for studying allele-specific regulatory dynamics. Here, we isolated adult fibroblasts (AFs) and placental fibroblasts (PFs) from mule tissues and reprogrammed them into induced pluripotent stem cells (iPSCs). Intriguingly, placental fibroblast-derived iPSCs (mpiPSCs) exhibited reduced reprogramming efficiency compared to adult fibroblast-derived iPSCs (maiPSCs). Through allele-specific expression (ASE) analysis, we systematically dissected transcriptional biases in parental cell types and their reprogrammed counterparts, revealing conserved preferential expression of asinine alleles in core pluripotency regulators (e.g., *POU5F1*/*OCT4*, *SOX2*, *NANOG*) across both cell lineages. Strikingly, mpiPSCs displayed stronger asinine allele dominance than maiPSCs, suggesting tissue-specific parental genomic imprinting. Mechanistic exploration implicated PI3K-AKT signaling as a potential pathway mediating the reprogramming inefficiency in placental fibroblasts. By integrating transcriptomic profiling with ASE technology, this study uncovers allele selection hierarchies during somatic cell reprogramming in hybrids and establishes a framework for understanding how parental genomic conflicts shape pluripotency establishment. These findings advance interspecies iPSC research by delineating allele-specific regulatory networks and providing insights into the molecular constraints of hybrid cellular reprogramming.

## 1. Introduction

As interspecific hybrids of horses (*Equus caballus*) and donkeys (*Equus asinus*), mules (*Equus mulus*) demonstrate distinct hybrid vigor, manifesting superior traits including enhanced musculoskeletal endurance, cognitive performance, adaptive thermogenesis, and innate immune competence compared to their parental species [1]. This phenotypic superiority, coupled with their obligate sterility stemming from diploid (2*n* = 63) chromosomal imbalance, positions mules as a unique biological system for investigating speciation barriers, meiotic recombination dynamics, and parent-of-origin genomic regulations [2,3]. The emergence of induced pluripotent stem cell (iPSC) technology has revolutionized comparative genomic studies in equids, enabling the derivation of pluripotent lineages from mule somatic tissues while bypassing ethical constraints associated with embryonic manipulation [4,5,6]. This approach facilitates unprecedented investigation of hybrid cellular physiology, epigenetic memory retention, and species-specific adaptation mechanisms. Nevertheless, fibroblast reprogramming efficiency remains constrained by intrinsic metabolic and proliferative limitations inherent to equid cells. Paradoxically, recent transcriptomic analyses reveal that mule-derived somatic cells achieve significantly higher reprogramming competency than those from either parental species, suggesting that hybrid genomes may harbor unique regulatory configurations that optimize pluripotency induction—a phenomenon challenging conventional assumptions about hybrid cellular plasticity [4]. These findings establish mule iPSCs as powerful tools for dissecting interspecies genomic interactions while providing unprecedented opportunities for conservation-oriented cellular engineering in endangered equids.

ASE analysis serves as a powerful tool for deconvoluting cis-regulatory architectures by quantifying transcriptional divergence between parental alleles within hybrid genomes [7,8,9]. In interspecific hybrids like mules, ASE profiling enables systematic identification of evolutionarily conserved regulatory elements, species-specific epigenetic modifications, and parent-of-origin effects governing cellular phenotypes [7].

In interspecies hybrids such as rice, and mules, the emergence of ASE patterns is orchestrated by an intricate interplay of regulatory mechanisms, encompassing parent-of-origin epigenetic modifications like differential DNA methylation at imprinting control regions [10,11]; cis-trans regulatory divergences arising from species-specific transcription factor binding affinities [12]; and structural genomic variations, including chromosomal rearrangements and copy number variations [13]. These multifaceted regulatory conflicts precipitate transcriptional disharmony between the horse and donkey genomes within mule cells, manifesting as coordinated suppression of metabolic pathways through H3K27me3-mediated silencing or stochastic activation of stress-responsive networks via enhancer hijacking [4]. During the process of iPSC reprogramming, such allele-level expression imbalances erect epigenetic barriers through the incomplete erasure of somatic chromatin states at imprinted loci and the asynchronous reactivation of species-specific enhancer–promoter interactions [9]. These molecular constraints ultimately influence reprogramming efficiency by modulating the fidelity of pluripotency network establishment, particularly impacting core regulators such as *OCT4* and *NANOG* through allele-specific chromatin accessibility patterns.

Placental fibroblasts serve as a uniquely powerful model system for elucidating allele-specific regulatory conflicts in interspecies hybrids as their evolutionarily conserved ICRs maintain ancestral parental allelic expression patterns through sustained methylation at gametic differentially methylated regions, a characteristic that distinguishes them from somatic fibroblasts which acquire tissue-restricted epigenetic states during differentiation [14]. Through establishing a comparative reprogramming research system based on mule placental and somatic fibroblasts we systematically elucidated how allelic expression imbalance affects nuclear reprogramming at the signaling pathway regulation level, specifically revealing the critical role of the PI3K-AKT signaling pathway in pluripotency remodeling, which is essential for stabilizing the pluripotency network. This research strategy reveals allele-specific differences between equine and asinine genomes at both placental and fibroblast levels and uncovers cross-species allelic expression patterns during cellular reprogramming.

In this study, using somatic and placental fibroblasts derived from mules as starting materials for reprogramming and employing our previously established induction system, we successfully generated iPSC lines from both mule somatic and placental fibroblasts. Furthermore, we conducted transcriptomic sequencing and ASE analysis on the fibroblasts and their corresponding iPSCs to explore the impact of the imbalanced expression of horse and donkey alleles on iPSC induction. This investigation not only enhances our understanding of how ASE in mule somatic cells influences their cellular biological functions but also provides critical insights into improving iPSC induction efficiency. These mechanistic insights not only redefine our understanding of hybrid cellular reprogramming but also yield an optimized protocol demonstrating a high efficiency gain over conventional methods, establishing foundational strategies for conservation-driven stem cell applications in endangered equids.

## 2. Materials and Methods

### 2.1. Reprogramming AFs and PFs to iPSCs

For this study reprogrammed cell lines from a reproductively capable mule, previously established in the laboratory, were utilized. Reprogramming studies were specifically conducted using fibroblast cell lines derived from the mule’s placenta and ear tissue [4].

Cells cultured in M10 medium (Supplementary Table S1) were dissociated at 70–80% confluence (~1.0 × 10^6^ cells per experiment) using TrypLE Select. For t-transfection, 0.25 million cells were seeded onto mitomycin-C-inactivated STO feeder layers in DOX-supplemented M15 medium (1.0 μg/mL; 631311, Clontech, Mountain View, CA, USA) using 10 cm dishes (Supplementary Table S1). The medium was refreshed every 48 h. Between days 10 and 14, emerging colonies were selected and maintained in the same DOX-containing medium. Reprogramming efficiency was determined by comparing colony formation in cultures treated with 2-deoxy-D-glucose (2-DG; D807272, MCE, Monmouth Junction, NJ, USA) or D-fructose-6-phosphate (F6P; MED21625, Medbio, Espoo, Finland) to untreated controls. Colonies expressing endogenous pluripotency markers (*OCT4*, *SOX2*, *NANOG*), validated by RT-qPCR, were propagated under DOX-free conditions to establish transgene-independent iPSC lines.

### 2.2. Culture Conditions of maiPSCs and mpiPSCs

To optimize culture conditions, DOX-dependent iPSCs derived from AFs and PFs, expressing *OCT4*, *SOX2*, and *NANOG*, were plated on feeder layers at a density of 1.2 × 10^4^ cells per well and maintained in M15 medium supplemented with DOX.

Building upon previously established mule iPSC culture protocols, a reprogramming system developed in our laboratory was employed [4]. The culture medium was enriched with small molecules and cytokines at the following final concentrations: CHIR99021 (GSK3 inhibitor), 3 μM; PD0325901 (MEK inhibitor), 1 μM; Gö6983 (PKC inhibitor), 5 μM; SP600125 (JNK inhibitor), 4 μM; SB203580 (P38 inhibitor), 10 μM; Y27632 (ROCK inhibitor), 10 μM; SB590885 (BRAF inhibitor), 0.5 μM; WH-4-023 (SRC inhibitor), 0.5 μM; XAV939, 5.0 μM; vitamin C, 50 μg/mL; LIF, 1000 U/mL; and Activin A, 20 ng/mL. The medium was replaced daily, and surviving cells were subcultured every two days. Endogenous pluripotency marker expression (*OCT4*, *SOX2*, and *NANOG*) was assessed after six days.

### 2.3. Culturing AFs, PFs, mpiPSCs, and maiPSCs

Distinct culture systems were employed for different cell types; adult fibroblasts (AFs) and placental fibroblasts (PFs) were expanded in M10 basal medium, whereas macaque- and porcine-induced pluripotent stem cells (designated as maiPSCs and mpiPSCs, respectively) required feeder layer support and underwent routine enzymatic passaging at 48–72-h intervals. The passaging procedure included: (1) medium removal followed by phosphate-buffered saline (PBS; Gibco 14, 190–144) rinsing; (2) dissociation using TrypLE Select (4 min incubation); (3) neutralization with K10 quenching medium, followed by mechanical disaggregation and cell pelleting via centrifugation (500× *g*, 3 min). Pelleted maiPSCs/mpiPSCs were resuspended in serum/LIF-supplemented maintenance medium for subculture. All procedures were conducted under standard mammalian cell culture conditions (5% CO_2_, 38.5 °C) unless otherwise specified.

For cryopreservation, cells at ~80% confluency were processed using freezing medium containing 90% fetal bovine serum (FBS) and 10% dimethyl sulfoxide (DMSO; D2650, Sigma, St. Louis, MO, USA). To ensure post-thaw viability, feeder-seeded 24-well plates (1.2 × 10^4^ cells/well) were prepared 48 h before cryorecovery.

### 2.4. AP Staining

Following seeding in 4-well culture plates, cellular specimens underwent sequential processing: initial PBS (1×) rinsing, fixation with 4% paraformaldehyde (P1110-100, Solarbio, Beijing, China) under ambient conditions (10 min), and post-fixation PBS washing. Alkaline phosphatase (AP) staining was subsequently initiated by applying a freshly formulated detection solution prepared through a three-stage protocol. First, sodium nitrite solution (50 μL) was combined with FRV alkaline solution (50 μL) for thermal activation (37 °C, 3 min). This pre-activated mixture then received sequential additions of ultrapure water (2.25 mL; W1503-500, Sigma, Saint Louis, MO, USA) and naphthol-As-BI alkaline solution (50 μL), followed by vortex-assisted homogenization. The resultant working solution was dispensed onto fixed cell monolayers for 16–18 h of dark-phase incubation to enable chromogenic development.

### 2.5. Karyotype

Following treatment with 0.2 μg/mL colchicine for 2.5 h in culture medium, cellular samples underwent enzymatic dissociation using TrypLE Select and were pelleted via centrifugation (1300 rpm, 3 min). The harvested cells were subjected to hypotonic shock through resuspension in 8 mL 0.075 M KCl (Sigma) and incubated at 37 °C for 40 min. Sequential fixation was performed by adding 1 mL methanol–glacial acetic acid (3:1) fixative to the suspension, followed by gentle agitation and centrifugation (1000 rpm, 10 min). After supernatant removal, the pellet underwent two iterative fixation cycles in fresh fixative (8 mL each), each including 37 °C incubation for 30 min. The final cell suspension (0.5 mL fixative) was dropped onto pre-chilled glass slides, oven-dried at 70 °C for 1 h, and stained with Giemsa solution (Sigma) for 10 min. Post-staining slides were rinsed with distilled water, air-dried, and analyzed via LUCIA Cytogenetics (Prague, Czechia).

### 2.6. In Vitro EB Formation Assay of maiPSCs and mpiPSCs

In vitro differentiation, maiPSCs and mpiPSCs were detached from culture dishes using TrypLE Select, resuspended in M10 medium, and then seeded into ultralow cell attachment U-bottom 96-well. After 3 days in suspension culture, EBs were transferred to gelatin-coated dishes and cultured for another 3 days prior to immunostaining.

### 2.7. Immunofluorescence Staining

Immunofluorescence specimens underwent sequential processing: PBS rinsing, fixation in 4% paraformaldehyde (10 min, RT), and permeabilization using PBS containing 0.1% Triton X-100/1% BSA (Sigma reagents, 30 min). Primary antibody incubation proceeded at 4 °C for 16–18 h, followed by three consecutive washes (5 min each) in permeabilization buffer. Secondary antibody exposure (RT, 1 h, dark phase) was followed by post-staining cleansing: one permeabilization buffer wash (5 min) and dual PBS rinses (5 min each). Chromatin counterstaining employed DAPI (C02-04002,Bioss, Woburn, MA, USA), with confocal imaging executed on the Olympus FLUOVIEW FV1000 platform (Tokyo, Japan). Antibody specifications are cataloged in Supplementary Table S2.

### 2.8. RT-qPCR Analysis

Total RNA extraction from cultured cells was performed using a RNeasy Mini Kit (74104, Qiagen, Duesseldorf, Germany). Synthesized complementary DNA (cDNA) was generated with the HiScript Q RT SuperMix (Vazyme R223-01) under qPCR-grade conditions. Target-specific primer pairs (Supplementary Table S3) were employed for RT-qPCR amplification conducted on an Applied Biosystems Veriti 96-Well Thermal Cycler, utilizing a KAPA SYBR FAST Universal qPCR Kit (KK4601, Roche-KAPA, Basel, Switzerland). Transcript quantification involved normalization to GAPDH reference values, with fold-change calculations executed via the ΔΔCt algorithm. Experimental data are expressed as mean ± SD from technical triplicate.

### 2.9. RNA Library Preparation and Sequencing

Total RNA was isolated from cellular specimens using a RNeasy Mini Kit (Qiagen) per the manufacturer’s guidelines. RNA integrity assessment via a Fragment Analyzer (Advanced Analytical) preceded poly(A)+ mRNA enrichment (1–2 μg input RNA) with the NEBNext Poly(A) mRNA Magnetic Isolation Module. Libraries were constructed using a NEBNext Ultra-RNA Library Prep Kit (E7770L, Beverly, MA, USA), followed by pooling and paired-end sequencing (150-bp reads) on an Illumina NovaSeq 6000 platform (San Diego, CA, USA) with an average sequencing depth of 10× per cellular sample.

### 2.10. Allele-Specific Expression Analysis

To calculate allele-specific expression for mule cells deriving from horse and donkey, we first used STAR (version 2.7.11) for alignment with the following parameters:--twopassMode Basic --genomeLoad NoSharedMemory --outSAMprimaryFlag AllBestScore --outSAMmultNmax −1 --outFilterMismatchNmax 0 --outSAMattrIHstart 0 --outReadsUnmapped Fastx --outSAMattributes NH HI AS nM NM MD jM jI MC [15]. Next, based on the mapping quality (mapq) in the generated SAM file, we determined whether each read aligned to the horse or donkey reference genome and then used a read ID file to split and reconstruct FASTQ files according to their respective reference genomes. We then realigned these source-specific FASTQ files separately to their respective references, sorted and converted the resulting SAM files to BAM files using SAMtools (V1.21) [16], and finally performed expression quantification with StringTie (V2.2.3) [17]. This yielded two separate allele-specific expression matrices, which were used in the subsequent analyses. Based on the quantitative results, they were converted to fragments per kilobase of transcript per million mapped reads (FPKM) for differential analysis. Differential genes were filtered with the thresholds of |log2 fold change| > 1 and *p*_value < 0.05, and the differential analysis tool used was edgeR (v3.42.4).

### 2.11. Data Visualization Functional Enrichment Analysis

Visualization pipelines employed ggplot2 (v1.0.12) for bar/bubble plots and pheatmap (v3.3.5) for gene cluster heatmaps. Functional enrichment analyses encompassed Gene Ontology (GO) and KEGG pathway assessments using clusterProfiler (v4.1.0) [18]. Gene set prioritization via GSEA (Broad Institute v4.0.3) [19].

## 3. Results

### 3.1. Establishment and Characterization of Induced Pluripotent Stem Cells (iPSCs) Derived from Mule Adult and Placental Fibroblasts

**Figure 1:**
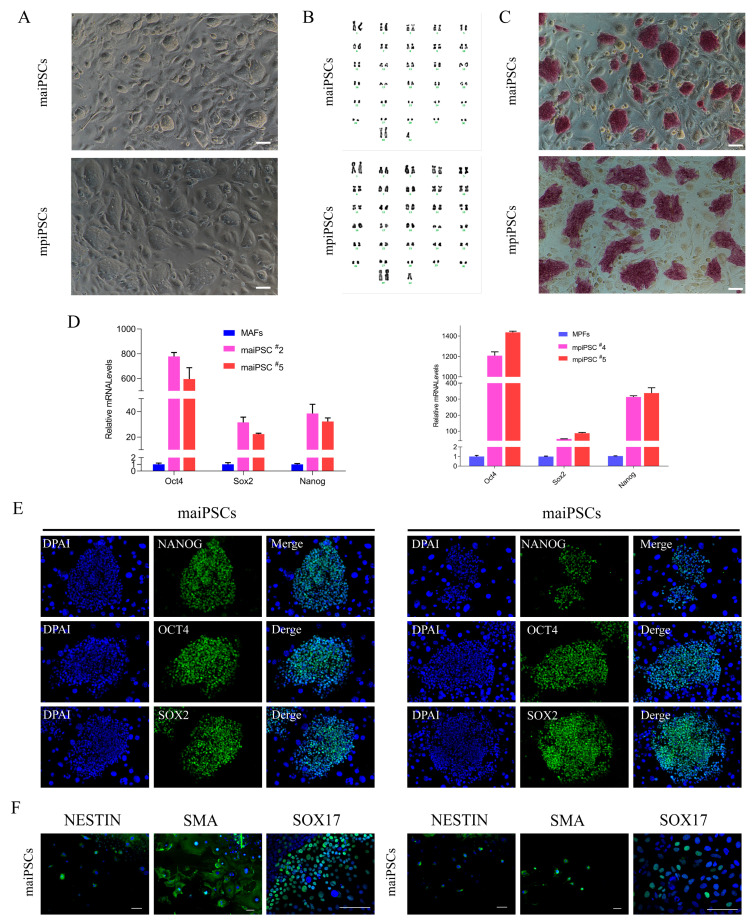
Characterization and pluripotency validation of induced pluripotent stem cells (iPSCs) derived from adult and placenta fibroblasts. (**A**) Morphological comparison of fibroblasts derived from adult and placental tissues (scale bars, 100 μm). (**B**) Karyotype analysis indicates normal chromosome numbers and structures in adult and placental fibroblasts. (**C**) Alkaline phosphatase staining demonstrates strong positive activity in maiPSCs and mpiPSCs, indicating pluripotent status (scale bars, 100 μm). (**D**) Quantitative real-time PCR analysis showing significantly increased expression levels of pluripotency markers (*NANOG*, *OCT4*, and *SOX2*) in maiPSCs and mpiPSCs compared to control fibroblasts. (**E**) Immunofluorescence staining confirms the expression of key pluripotency proteins *NANOG* (green), *OCT4* (green), and *SOX2* (green) in maiPSCs and mpiPSCs. DAPI (blue) indicates nuclear staining (scale bars, 100 μm). (**F**) Immunofluorescence analysis of differentiated cells derived from maiPSCs and mpiPSCs demonstrates expression of lineage-specific markers including NESTIN, SMA, and SOX17, indicating differentiation potential into all three germ layers (scale bars, 100 μm).

To quantitatively evaluate the reprogramming potential of AFs and placental fibroblasts PFs, we introduced six doxycycline-inducible reprogramming factors (pOMSK and hRL) via piggyBac transposition. Following transfection, colony formation was monitored over time (Table 1, Figure 1A). At day 10, the mean number of alkaline phosphatase-positive (AP+) iPSC colonies generated from adult fibroblasts (AFs1# and AFs2#) was 105.5 ± 27.6 (*n* = 2), compared to 3.3 ± 2.9 (*n* = 3) from placental fibroblast lines (PFs1#, PFs2#, PFs3#) (*p* < 0.01, unpaired *t*-test). The difference remained significant at day 14 (AFs: 146.5 ± 51.5; PFs: 12.3 ± 7.8, *p* < 0.01) and day 18 (AFs: 193.0 ± 38.2; PFs: 18.7 ± 6.8, *p* < 0.001).

To assess clonal stability after expansion, we picked colonies at day 10–15 and propagated them as single-cell clones for at least two passages. After two passages, the cloning efficiency (percentage of initial colonies giving rise to stably expandable iPSC lines) was 95.8% (229/239) for maiPSC lines and 75% (27/36) for mpiPSC lines. Both cell types were stably maintained in an undifferentiated state on STO feeder layers for over 30 passages, retained a normal karyotype, and were designated as mule adult iPSCs (maiPSCs) and mule placental iPSCs (mpiPSCs), respectively (Figure 1A,B). Alkaline phosphatase (AP) staining confirmed the pluripotent status of both maiPSCs and mpiPSCs, which also expressed high levels of key endogenous pluripotency markers (Figure 1C,D). Immunofluorescence analysis further revealed comparable expression patterns of *OCT4*, *SOX2*, and *NANOG* in both cell types (Figure 1E). To assess their differentiation potential, embryoid body (EB) formation assays were performed in vitro. Immunostaining demonstrated that both maiPSCs and mpiPSCs were capable of differentiating into derivatives of all three germ layers (Figure 1F). These findings confirm that both maiPSCs and mpiPSCs exhibit typical molecular and functional characteristics of pluripotent stem cells.

### 3.2. Allele-Specific Expression Data Processing of Mule Transcriptomes

**Figure 2:**
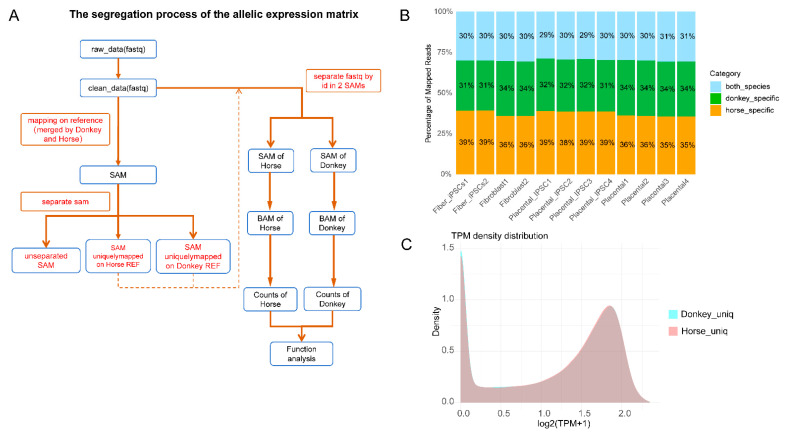
Pipeline and characterization of allelic expression analysis between donkey and horse genomes. (**A**) Workflow illustrating the segregation process used for generating allelic expression matrices. (**B**) The proportion of mapped RNA-seq reads is classified into three categories: horse-specific (green), donkey-specific (orange), and shared between species (“both species,” blue). The percentages are consistent across different cell types and tissues, highlighting stable allelic expression ratios. (**C**) Density plot illustrating transcript abundance distributions (log2 (TPM + 1)) for donkey-specific (cyan) and horse-specific (pink) uniquely mapped reads.

To investigate whether the lower reprogramming efficiency of mule placental fibroblasts is influenced by allele-specific regulation, we performed transcriptomic sequencing on four types of mule cells: AFs, PFs, maiPSCs, and mpiPSCs (Figure 2A). Subsequently, we employed an allele-specific expression analysis pipeline based on STAR software to process the mule transcriptomic data [15]. As shown in Figure 2B, the analysis revealed that the proportion of mapped reads originating from horses, donkeys, and shared sources in the mule transcriptome was approximately 1:1:1. The shared reads likely result from the high genomic similarity between horses and donkeys.

Next, we utilized reads that were specifically mapped to either the horse or donkey genome for realignment, annotation, and quantification. This process generated allele-specific expression matrices for horse- and donkey-derived alleles. Based on homologous gene analysis, we harmonized or filtered homologous genes expressed in horse and donkey alleles (Figure 2C). Ultimately, we identified 15,150 common alleles across the mule transcriptome.

### 3.3. Differences in Allele-Specific Expression Between Fibroblasts and Their Reprogrammed iPSCs

**Figure 3:**
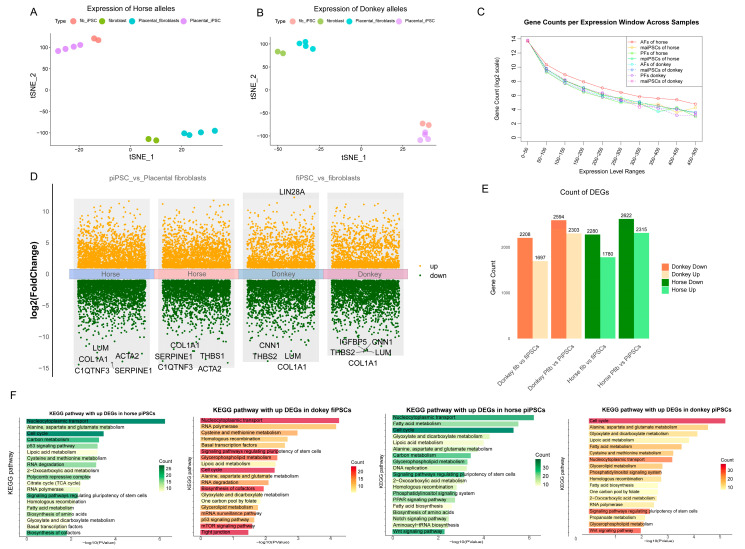
Analysis of allele-specific gene expression and functional enrichment between horse and donkey genomes. (**A**,**B**) t-SNE visualization based on allele-specific expression of horse (**A**) and donkey (**B**) alleles (**C**). Gene count distribution across different expression level windows (log-scaled TPM ranges) for horse- and donkey-specific transcripts. (**D**) Volcano plots illustrating differential gene expression analysis comparing induced pluripotent stem cells (mpiPSCs vs. PFs and maiPSCs vs. AFs). Upregulated genes are marked in orange, downregulated genes in green. (**E**) Counts of significantly upregulated and downregulated DEGs in donkey- and horse-derived iPSCs compared to fibroblasts. (**F**) KEGG pathway enrichment analysis of significantly upregulated DEGs in horse- and donkey-derived maiPSCs and mpiPSCs.

After obtaining the ASE matrix for the four cell types, we focused on comparing the expression differences between different fibroblast types and their corresponding reprogrammed iPSCs. ASE analysis revealed a pronounced bias toward horse-derived allele expression in placental fibroblasts; however, this allele expression bias disappeared after reprogramming into iPSCs (Figure 3A,B).

We compared the gene expression differences between the two fibroblast types and their corresponding iPSCs (Figure 3C,D). In both iPSCs, regardless of horse- or donkey-derived alleles, the upregulated genes were enriched in pathways related to pluripotency regulation, cell cycle, and nucleocytoplasmic transport, which align with the characteristics exhibited during iPSC reprogramming (Figure 3E). In fibroblasts, however, the differentially expressed alleles were primarily involved in coenzyme biosynthesis, the TCA cycle, mTOR signaling, and tight junction pathways. In the lower-efficiency mpiPSCs, pathways such as PPAR signaling, WNT signaling, phosphatidylinositol signaling, and Notch signaling were found to participate in the reprogramming process (Figure 3F). These differences in allele-specific expression reflect how the two types of fibroblasts influence reprogramming efficiency through distinct regulatory mechanisms during the reprogramming process.

### 3.4. Comparative Analysis of Horse and Donkey Alleles in Mule iPSCs

**Figure 4:**
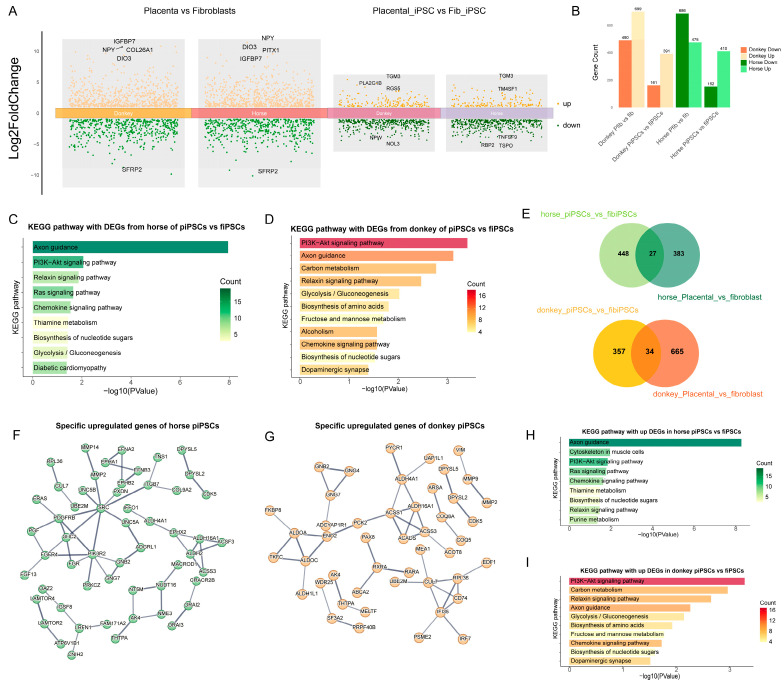
Differential gene expression and functional pathway enrichment analysis in mpiPSCs compared to maiPSCs**.** (**A**) Volcano plots representing DEGs identified between AFs vs. PFs and maiPSCs vs. mpiPSCs. Orange represents the upregulated genes in the corresponding comparison groups, and green represents the downregulated genes in the comparison groups. The differential genes were filtered using the thresholds of |log2FC| > 1 and pvalue < 0.05. (**B**) Summary of DEG counts showing species-specific (horse and donkey) upregulated and downregulated gene numbers in a comparison of donkey AFs vs. PFs and maiPSCs vs. mpiPSCs. (**C**,**D**) KEGG pathway enrichment analysis for DEGs in horse (**C**) and donkey (**D**) maiPSCs compared with mpiPSCs. (**E**) Venn diagrams illustrating the overlap of DEGs between maiPSCs vs. mpiPSCs and AFs vs. PF in horses and donkeys, revealing common and unique gene sets. (**F**,**G**) Protein–protein interaction network analysis of specific upregulated genes identified in horses (**F**) and donkeys (**G**) in mpiPSCs. (**H**, **I**) Additional KEGG pathway analyses highlighting enrichment differences between horses (**H**) and donkeys (**I**) in mpiPSCs.

To further explore the regulatory mechanisms by which horse and donkey alleles influence mpiPSCs reprogramming, we compared differences of ASE across the two types of fibroblasts and their respective iPSCs (Figure 4A). As shown in the figure, the number of differentially expressed alleles was significantly reduced following reprogramming in both types of iPSCs (Figure 4B). Further analysis revealed that horse- and donkey-derived alleles enriched pathways such as PI3K-AKT signaling, Relaxin signaling, and Axon guidance in both types of iPSCs (Figure 4C,D). Considering that partial differences in allele expression may be inherited from somatic cells, we filtered out fibroblast-specific differential alleles to exclude interspecies differences and obtained a gene set specifically responsible for iPSC-specific differences (Figure 4E). Using these refined gene sets, we constructed regulatory networks for horse and donkey alleles (Figure 4F,G). Enrichment analysis of the filtered genes revealed significant enrichment in pathways such as PI3K-AKT signaling and chemokine signaling (Figure 4H,I). These results indicate that at the transcriptional level the primary differences between the two types of iPSCs are reflected in pathways such as PI3K-AKT signaling and chemokine signaling.

### 3.5. Potential Regulatory Mechanisms of Horse and Donkey Alleles in mpiPSCs

**Figure 5:**
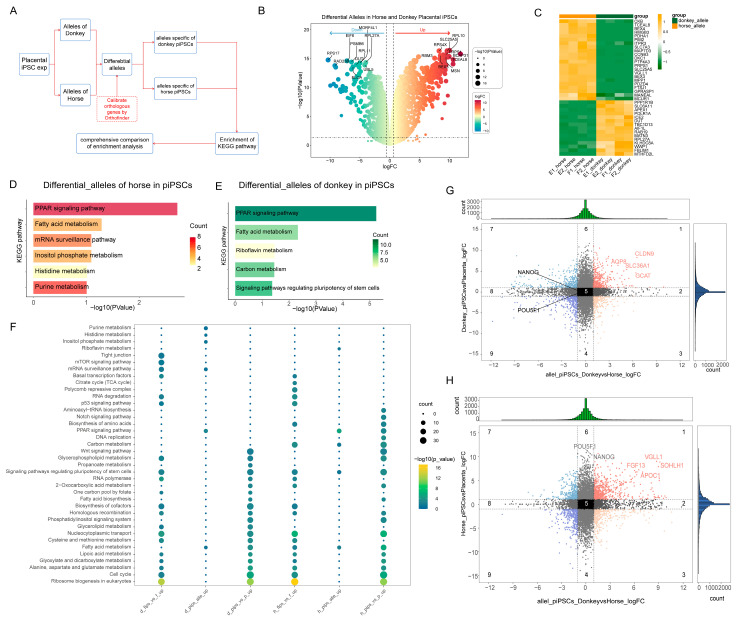
Comparative analysis of differential allele-specific expression and functional enrichment between donkey and horse alleles in mpiPSCs. (**A**) Workflow diagram illustrating the pipeline for identifying allele-specific differential gene expression between donkey and horse alleles in mpiPSCs. (**B**) Volcano plot highlighting significantly differentially expressed alleles (DEAs) between alleles in mpiPSCs, with representative significantly upregulated and downregulated alleles labeled. (**C**) Heatmap of selected DEAs illustrating clear clustering between donkey-allele-dominated and horse-allele-dominated gene expression patterns. (**D**,**E**) KEGG pathway enrichment analyses showing significantly enriched biological pathways in horse (**D**) and donkey (**E**) differential alleles, highlighting distinct metabolic and pluripotency regulatory pathways between species. (**F**) KEGG pathway enrichment across different allele-specific comparisons. (**G**,**H**) Scatterplots comparing allele-specific expression fold-changes between alleles in mpiPSCs, with notable pluripotency-related and lineage-specific genes highlighted. Dark blue, red and yellow dots in the figure represent genes with significant DEGs in the groups, while the black and gray dots represent non-differential genes.

Given the presence of allele-specific expression bias, we further explored the interactions between horse- and donkey-derived alleles within iPSCs (Figure 5A). After pairing and normalizing homologous genes, we performed differential expression analysis on horse- and donkey-derived alleles in mpiPSCs (Figure 5B,C). The results revealed that certain genes associated with the mRNA surveillance pathway exhibited higher expression trends in horse-derived alleles, while riboflavin metabolism showed elevated expression in donkey-derived alleles (Figure 5D,E).

Subsequent enrichment analysis for the two sets of alleles revealed that the expression of horse-derived alleles was related to RNA signaling pathways and histidine metabolism (Figure 5F). In contrast, the expression of pluripotency-related genes in iPSCs was primarily enriched in donkey-specific differential alleles. By comparing these results with previous differential gene enrichment findings, we further confirmed that donkey-derived alleles play a dominant role in the regulation of pluripotency in mpiPSCs. Common pluripotency-regulating genes, such as *NANOG* and *OCT4*, were more significantly expressed in donkey-derived alleles (Figure 5G,H). The findings indicate that in mpiPSCs the expression of donkey-derived alleles remains significantly suppressed, which impacts the regulation of pluripotency during the reprogramming process. This suppression may represent a critical factor contributing to the lower reprogramming efficiency of placental fibroblast-derived cells.

### 3.6. Regulation of PI3K-AKT Signaling Pathway in Mule Placental Cell Reprogramming to iPSCs

**Figure 6:**
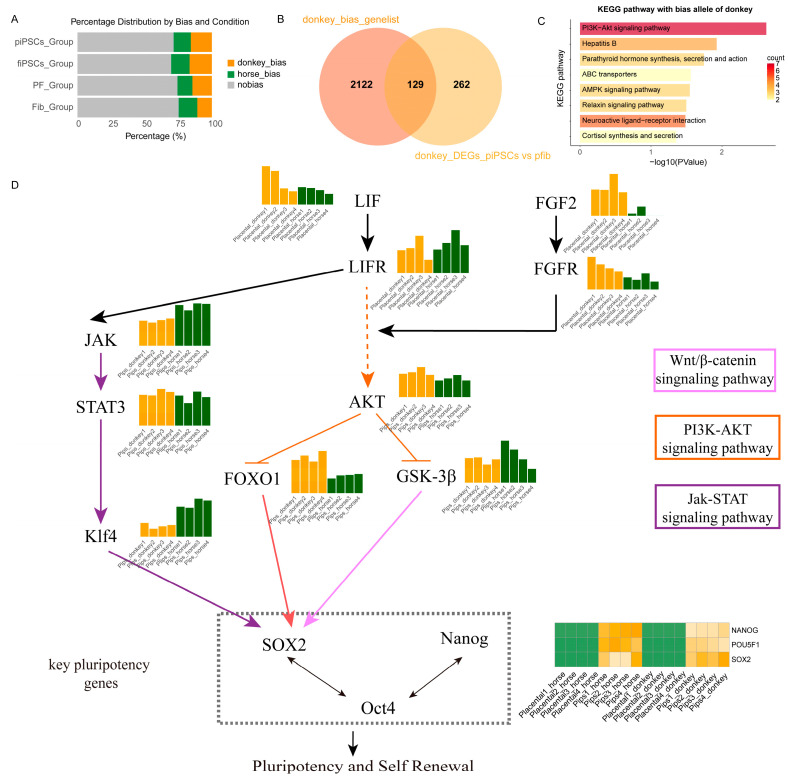
Allelic bias and signaling pathway analysis associated with pluripotency and self-renewal in donkey and horse piPSCs. (**A**) Distribution of allelic expression bias in mpiPSCs, maiPSCs, PFs, and AFs groups. (**B**) Venn diagram comparing donkey-biased genes and donkey-derived DEGs identified specifically in mpiPSCs. (**C**) KEGG pathway enrichment analysis showing significantly enriched pathways among donkey-biased alleles. (**D**) Integrated signaling network schematic illustrating key signaling pathways (JAK-STAT, PI3K-AKT, and Wnt/β-catenin) contributing to pluripotency and self-renewal. Central pluripotency regulators (*SOX2*, *OCT4*, and *NANOG*) and their upstream activators and downstream effectors are indicated, emphasizing intricate signaling crosstalk governing stem cell maintenance.

By comparing the differences of ASE in two fibroblasts and their respective reprogrammed iPSCs, we observed that donkey-derived alleles had a more prominent impact on pluripotency in mule placental fibroblasts and their reprogrammed mpiPSCs (Figure 6A). To precisely identify the factors contributing to the lower reprogramming efficiency of placental cells, we filtered the gene sets influencing reprogramming. A detailed evaluation of allele expression bias in the four cell types confirmed earlier conclusions that donkey alleles dominate expression during the reprogramming process (Figure 6B). Subsequently, we extracted genes exhibiting expression bias in mpiPSCs and integrated them with differentially expressed genes, identifying a common gene set of 129 genes for enrichment analysis. The results revealed significant enrichment of the PI3K-AKT signaling pathway (Figure 6C).

To systematically elucidate the role of the PI3K-AKT pathway, we constructed a regulatory network illustrating how the pathway influences pluripotency regulation in mpiPSCs. This network explains how PI3K-AKT inhibits the reprogramming process in placental fibroblasts, resulting in the lower efficiency observed in mpiPSCs induction (Figure 6D).

## 4. Discussion

Efficient iPSC reprogramming is influenced by factors such as cellular metabolism, epigenetic regulation, and cell cycle dynamics [20,21,22]. In hybrid animal cells, how these factors interact with allele-specific expression to affect reprogramming efficiency remains unclear [23]. Our results demonstrate that maiPSCs exhibit higher reprogramming efficiency than mpiPSCs, as evidenced by two key findings: (1) more iPSC colonies formed by day 14 of reprogramming; (2) higher colony establishment efficiency after two passages. This discrepancy may be attributed to the unique properties of placental fibroblasts. Using RNA-seq analysis and allele-specific expression profiling, we identified key factors influencing the lower reprogramming efficiency of mpiPSCs.

Although hybrid animal somatic cells have been shown to reprogram successfully into iPSCs in vitro, the advantages of hybrid vigor in mule iPSCs remain underexplored [4]. In this study, by comparing mule placental fibroblasts and somatic fibroblasts we investigated the factors affecting mule iPSC reprogramming efficiency through allele-specific expression analysis. Based on RNA-seq data, we confirmed that the PI3K-AKT signaling pathway plays a critical role in the reprogramming of placental fibroblasts. The imbalance in allele-specific expression presents a barrier to achieving pluripotency. In contrast, maiPSCs displayed better adaptability in balancing allele-specific expression, which could explain their higher reprogramming efficiency. This phenomenon may be linked to epigenetic factors, as the demethylation process during reprogramming can alleviate the effects of methylation or genomic imprinting on transcriptional expression in placental cells [24].

Furthermore, both AFs and PFs exhibited stable expression of highly activated endogenous pluripotency genes after reprogramming, indicating that mule iPSCs maintain genomic stability post-reprogramming. By comparing the allele-specific expression levels between mpiPSCs and maiPSCs, we identified 412 horse-specific differentially expressed alleles and 379 donkey-specific differentially expressed alleles which are involved in the PI3K-AKT signaling pathway and play a significant role in determining the reprogramming efficiency of mpiPSCs. Additionally, after filtering for allele-specific differences between placental and somatic fibroblasts these genes were found to be precise targets involved in epigenetic regulation in mule placental cells, under the control of genomic imprinting mechanisms.

Interestingly, during the reprogramming process maiPSCs exhibited significant activation of donkey-derived alleles, which could contribute to the higher efficiency of fibroblast reprogramming. In contrast, in placental fibroblasts donkey-derived alleles showed greater activity and were more likely to participate in placental-specific functions, such as immune regulation and maintenance of the maternal–fetal interface. These roles may limit their ability to reprogram into a pluripotent state.

In conclusion, this study highlights the differences in allele-specific expression between mule somatic and placental fibroblasts and their impact on reprogramming efficiency. The specific expression of horse and donkey alleles in different cell types largely determines the activation efficiency of pluripotency genes and the ultimate formation of iPSCs. These findings indicate that allele-specific expressions during reprogramming are influenced by multiple factors, including cell origin, reprogramming factors, and epigenetic status. This study provides new insights into the molecular mechanisms of reprogramming in mule cells and identifies potential molecular targets to improve reprogramming efficiency. Future research should further investigate the regulatory mechanisms of allele-specific expression during reprogramming, particularly how modulating allele-specific expression could enhance reprogramming efficiency.

## 5. Conclusions

This study successfully established and characterized induced pluripotent stem cells (iPSCs) from both adult and placental fibroblasts of mules, revealing notable differences in reprogramming efficiency. Mule adult fibroblasts exhibited higher reprogramming efficiency than placental fibroblasts, despite both iPSC types displaying comparable pluripotency markers and differentiation capacity. Through allele-specific expression (ASE) analysis, we observed a pronounced allele expression bias in placental fibroblasts favoring horse-derived alleles, which was alleviated after reprogramming. In mule placental iPSCs (mpiPSCs), although donkey-derived alleles were relatively enriched in pluripotency-related genes their expression appeared suppressed. Notably, this suppression was particularly evident in genes associated with the PI3K-AKT signaling pathway and may contribute to the lower reprogramming efficiency observed in placental fibroblasts. However, we acknowledge that our data are correlative in nature and that direct functional validation will be required to establish a causal relationship between PI3K-AKT pathway activity and reprogramming outcomes. Furthermore, our study is limited by the number of biological replicates and the absence of additional cell types from donkey and horse, which should be addressed in future investigations to strengthen and generalize our findings. Taken together, our results highlight the potential importance of allele-specific regulatory mechanisms in influencing iPSC reprogramming efficiency and provide a foundation for future research into the molecular basis underlying interspecies differences in pluripotency regulation.

## Figures and Tables

**Table 1:** Number of iPSC colonies generated from mule adult and placental fibroblasts at different time points.

Cell Lines	No. of iPSCs Colonies at Day 10	No. of iPSCs Colonies at Day 14	No. of iPSCs Colonies at Day 18
AFs1#	125	183	220
AFs2#	86	110	166
PFs1#	0	4	12
PFs2#	5	19	25
PFs3#	5	14	19

## Data Availability

The RNA-seq data generated in this study are available only on request. Additional data generated during this study can be obtained upon request from the corresponding author.

## Supplementary Materials

The following supporting information can be downloaded at: https://www.mdpi.com/article/10.3390/cimb47080671/s1.
