# Supplementary material for: Comparative Study of Reprogramming Efficiency and Regulatory Mechanisms of Placental- and Fibroblast-Derived Induced Pluripotent Stem Cells (iPSCs) in Mules
Source: Curr Issues Mol Biol. 2025 Aug 19;47(8):671. doi: 10.3390/cimb47080671 (PMC12384536; doi:10.3390/cimb47080671)
Supplement: Supplementary file 1 [file cimb-47-00671-s001.zip › cimb-3749144-supplementary.pdf]

Table S1. Medium used for cells culture

M10 medium

M10 medium	Supplier	Catalog number
Knockout DMEM	Gibco	10829-018
1×Penicillin-Streptomycin	Gibco	15140-122
1×GlutaMAX	Gibco	35050-061
1×MEM Non-Essential Amino Acids	Gibco	11140-050
15% FBS	BI	04-002-1A

M15 medium

M15 medium	Supplier	Catalog number
Knockout DMEM	Gibco	10829-018
1×Penicillin-Streptomycin	Gibco	15140-122
1×GlutaMAX	Gibco	35050-061
1×MEM Non-Essential Amino Acids	Gibco	11140-050
0.1 mM 2-mercaptoethanol	Sigma	M6250
LIF	Millipore	LIF1010
Vitamin C	Sigma	49752
15% FBS	BI	04-002-1A

Table S2. Antibodies in this study

Antibodies	Antibody type	Dilution	Supplier<br>Catalog number
Oct-3/4	Mouse Monoclonal antibody IgG	1:200	Santa Cruz Biotechnology sc-5279
Sox2	Rabbit polyclonal Antibody IgG	1:200	Millipore AB5603
Nanog	Rabbit polyclonal Antibody IgG	1:50	Abcam ab80892
NESTIN	Rabbit polyclonal Antibody IgG	1:100	BOSTER BA1289
α-SMA	Mouse Monoclonal antibody IgG	1:200	R and D Systems MAB1420
SOX17	Goat Polyclonal antibody IgG	1:200	R and D Systems AF1924
Donkey anti-Rabbit IgG (H+L) Highly Cross-Adsorbed Secondary Antibody, Alexa Fluor® 488	Rabbit polyclonal Antibody IgG	1:500	ThermoFisher A21206
Donkey anti-Mouse IgG (H+L) Highly Cross-Adsorbed Secondary Antibody, Alexa Fluor® 488	Mouse polyclonal Antibody IgG	1:500	ThermoFisher A21202
Donkey anti-Goat IgG (H+L) Highly Cross-Adsorbed Secondary Antibody, Alexa Fluor® 488	Goat polyclonal Antibody IgG	1:500	ThermoFisher A11055
Donkey anti-Rat IgG (H+L) Highly Cross-Adsorbed Secondary Antibody, Alexa Fluor 488	Rat polyclonal Antibody IgG	1:500	Invitrogen A21208

Table S3. Primers used in this study.

Target	Forward primer (5'-3')	Reverse primer (5'-3')
GAPDH	CCACCCCTAACGTGTCAGTC	AAAGTGGTCGTTGAGGGCAA
OCT4	GGGGTCCTCACTTCACTACG	AACTTCACCTTCCCTCCAACC
SOX2	CACCCACAGCAAATGACAGC	AGTTTTCTTGTCGGCATCGC
NANOG	TCATCCACCAGTCCCAGAGT	TGTAGTTGCTGAGCCCCGA
